# Niche partitioning of avian predators in northern grasslands amended by biosolids

**DOI:** 10.1002/ece3.7461

**Published:** 2021-05-01

**Authors:** Arianna E. C. Ormrod, Francis I. Doyle, Kirstie J. Lawson, Karen E. Hodges

**Affiliations:** ^1^ Department of Biology University of British Columbia Okanagan Kelowna BC Canada; ^2^ Wildlife Dynamics Consulting Terrace BC Canada

**Keywords:** *Asio flammeus*, *Asio otus*, biosolids, British Columbia, *Circus hudsonius*, corvids, diet, Long‐eared Owl, Northern Harrier, rangeland, Short‐eared Owl

## Abstract

Many food webs are affected by bottom‐up nutrient addition, as additional biomass or productivity at a given trophic level can support more consumers. In turn, when prey are abundant, predators may converge on the same diets rather than partitioning food resources. Here, we examine the diets and habitat use of predatory and omnivorous birds in response to biosolids amendment of northern grasslands used as grazing range for cattle in British Columbia, Canada. From an ecosystem management perspective, we test whether dietary convergence occurred and whether birds preferentially used the pastures with biosolids. Biosolids treatments increased Orthoptera densities and our work occurred during a vole (*Microtus spp*.) population peak, so both types of prey were abundant. American Kestrels (*Falco sparverius*) consumed both small mammals and Orthoptera. Short‐eared Owls (*Asio flammeus*) and Long‐eared owls (*Asio otus*) primarily ate voles (>97% of biomass consumed) as did Northern Harriers (*Circus hudsonius,* 88% vole biomass). Despite high dietary overlap, these species had minimal spatial overlap, and Short‐eared Owls strongly preferred pastures amended with biosolids. Common Ravens (*Corvus corax*), Black‐billed Magpies (*Pica hudsonia*), and American Crows (*Corvus brachyrhynchos*) consumed Orthoptera, Coleoptera, vegetation, and only a few small mammals; crows avoided pastures with biosolids. Thus, when both insect and mammalian prey were abundant, corvids maintained omnivorous diets, whereas owls and Harriers specialized on voles. Spatial patterns were more complex, as birds were likely responding to prey abundance, vegetation structure, and other birds in this consumer guild.

## Graphical abstract

Biosolids are often applied to restore degraded terrestrial habitats. Nomadic owls and harriers nested in areas with biosolids applications and consumed voles as their main diet. Corvids consumed Orthoptera and vegetation, both of which were enhanced on areas with biosolids.

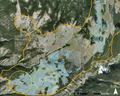

## 1 INTRODUCTION

Nutrient addition to terrestrial ecosystems may alter food web dynamics via bottom‐up effects on standing biomass and productivity at one or more trophic levels. Indeed, this property of fertilization has driven the use of biosolids as a restoration tool for degraded rangelands, as biosolids improve soils and increase forage production for cattle (Avery et al., 2019). Biosolids are the treated, stabilized, and sterilized remains left after wastewater treatment; they contain many macronutrients and micronutrients that enhance plant growth and help soils retain water (McFarland et al., 2010; Shammas & Wang, 2008). These effects are multi‐year. The biosolids themselves take several years to degrade, and as plants respond to nutrient addition, more litter is produced and incorporated back into soil.

Although these positive impacts of biosolids on soils and forage plants are well known, we do not know the extent to which these bottom‐up effects cascade through the ecosystem to predators. In British Columbia, Canada, grasslands have been negatively affected by habitat loss, fragmentation, over‐grazing, and pesticides; these threats have contributed to declines in many raptor species (IUCN Redlist, 2020; McClure et al., 2018; Poulin et al., 2001; Rosenberg et al., 2019). Here, we examine diets and habitat selection of birds of prey and corvids in a northern grassland to determine whether biosolids amendment enabled dietary convergence of specialist and generalist consumers on abundant prey, and to examine whether birds selected areas where biosolids had been applied.

**FIGURE 1:**
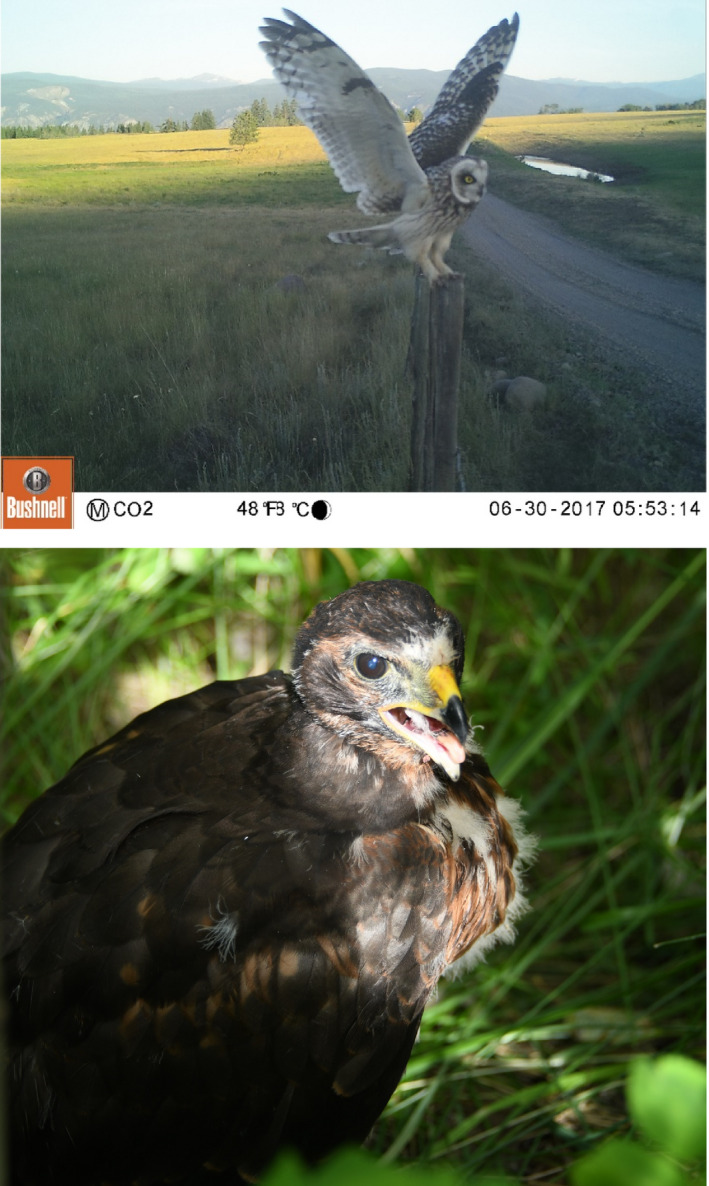
Images of two of the predatory birds that nested on the OK Ranch. (a) A Short‐eared Owl, using one of the many fenceposts separating pastures on the OK Ranch. We deployed wildlife cameras on some fenceposts to observe which raptors used fenceposts as perches. (b) A Northern Harrier chick, shortly before fledging. The nest was in a willow thicket. Photograph by KE Hodges

Biosolids dramatically improved plant growth and duration of growing season on our study area, extending the growing season by several weeks and often more than doubling biomass accumulation (Avery et al., 2019; Newman et al., 2014; Wallace et al., 2016). Further, pastures amended with biosolids supported more grasshoppers (Gaudreault et al., 2019) and songbirds (unpublished data). Our work occurred during a vole population peak in 2017, as confirmed by field observations from 2015 to 2020 of voles and vole sign and live‐trapping in 2019–2020 (Meineke, 2020). Several nomadic species bred on the study area (Figure 1), including Long‐eared Owls (*Asio otus*), Short‐eared Owls (*Asio flammeus*), and Northern Harriers (*Circus hudsonius*). Migratory American Kestrels (*Falco sparverius*) were common (Buers et al., 2019). These species joined the resident generalist corvids, American Crows (*Corvus brachyrhynchos*), Black‐billed Magpies (*Pica hudsonia*), and Common Ravens (*Corvus corax*).

Short‐eared Owls and Long‐eared Owls coexist across the Holarctic, with both species mainly eating small mammals (Birrer, 2009; González‐Rojas et al., 2017; Selçuk et al., 2017), but diets varying across their range. Within grassland areas, diets of Northern Harriers are poorly known, but they may focus on voles when these prey are abundant (Doyle & Smith, 2001; Schimpf et al., 2020; Smith et al., 2011). Limited work shows that corvids are generalists and scavengers. Magpies eat Carabid beetles, Orthoptera, carrion, seeds, and small mammals (Trost, 1999), as do crows (Annala et al., 2012; Kennedy & Otter, 2015; Verbeek & Caffrey, 2002). Ravens have diverse diets, and regularly consume voles and other small vertebrates (Boarman & Heinrich, 1999; Temple, 1974).

Within this grassland ecosystem, the birds of prey and corvids could respond in a number of ways to the presence of biosolids‐amended pastures. Birds could respond to the induced changes in vegetative structure, prey, or in reaction to presence of other birds, but it is not known for any of these species whether they select or avoid biosolids‐amended pastures. Further, Northern Harriers and Short‐eared owls are ground‐nesters, whereas the others are tree‐nesters and would need to find nest trees near to suitable hunting sites; for all species, territorial defense of nests suggests spatial segregation is likely.

For this community of predatory and omnivorous birds in a prey‐rich nutrient‐amended environment, our major objectives are to (a) examine whether birds disproportionately used pastures that had been amended with biosolids, (b) determine diet composition for these species, and (c) assess dietary niche breadths. We expected birds would preferentially use biosolids‐amended pastures, and that bird diets would converge on the common abundant prey. Specifically, we predicted owls and harriers would consume primarily voles, whereas corvids would consume both Orthoptera and voles. We also expected to see spatial separation of owl nests across the Ranch, in keeping with territorial defense.

## 2 METHODS

The study took place on the 45.3 km^2^ upland portion of the OK Ranch, a cattle ranch located northwest of Clinton, British Columbia. The ranch has a dry, mild climate with an average annual rainfall of 401 mm and an average annual temperature of 3°C. It is an arid grassland ecosystem with a few small lakes and marshes. There are small, isolated, and open forest stands of Douglas fir (*Pseudotsuga menziesii*) and aspen (*Populus tremuloides*). The most common shrubs are juniper (*Juniperus communis*), snowberry (*Symphorocarpis albus*), and soapberry (*Shepherdia canadensis*). The dominant grasses are bluebunch wheatgrass (*Pseudoroegneria spicata*), needle‐and‐thread grass (*Hesperostipa comata*), junegrass (*Koeleria macrantha*), and Nevada bluegrass (*Poa secunda juncifolia*). Common flowering plants are pussytoes (*Antennaria umbrinella* and *A. dimorpha*) and prairie sagewort (*Artemisia frigida*).

Since 2014, biosolids have been applied as a soil amendment. During 2014–2016, biosolids were spread by pull‐spreaders on 11.1 km^2^ (24.6%) of the study area, at a rate of 57,000 kg/ha (unpublished data, SYLVIS Environmental Ltd.). We documented ~3.8 fold increases in grasshopper densities 1–2 years postapplication of biosolids compared with untreated sites (Gaudreault et al., 2019). There was a distinct and large peak in vole numbers in 2017, detected via incidental observations of voles, runways, and grass clippings during our fieldwork every summer from 2015 to 2020. Specifically, in 2017, we routinely saw voles and vole runways while doing other fieldwork, whereas in other years such observations were scarce. During 2019 and 2020, live‐trapping indicated voles were rare (Meineke, 2020; Hodges and Doyle unpublished data), consistent with there having been a peak in 2017 followed by a decline and low.

### 2.1 Observations of birds and nests

From mid‐April to August 2017, we collected “seen sheet” data for a number of bird and mammal species (Hochachka et al., 2000; Poulin et al., 2001). We mapped the study area into 75 pastures or forested areas, using roads, fences, ponds, and history of biosolids applications as boundaries; biosolids applications were mapped as GIS layers by SYLVIS Environmental Ltd. (unpublished data), but applications also followed natural boundaries and were thus obvious when in the field. As we drove, we recorded when and where we first saw each species of interest; observations occurred between 0500 and 2200 hr. Because pastures, woodlands, and areas with biosolids were clearly separated by natural boundaries, we could easily assign each individual animal to the patch in which it was first observed.

**FIGURE 2:**
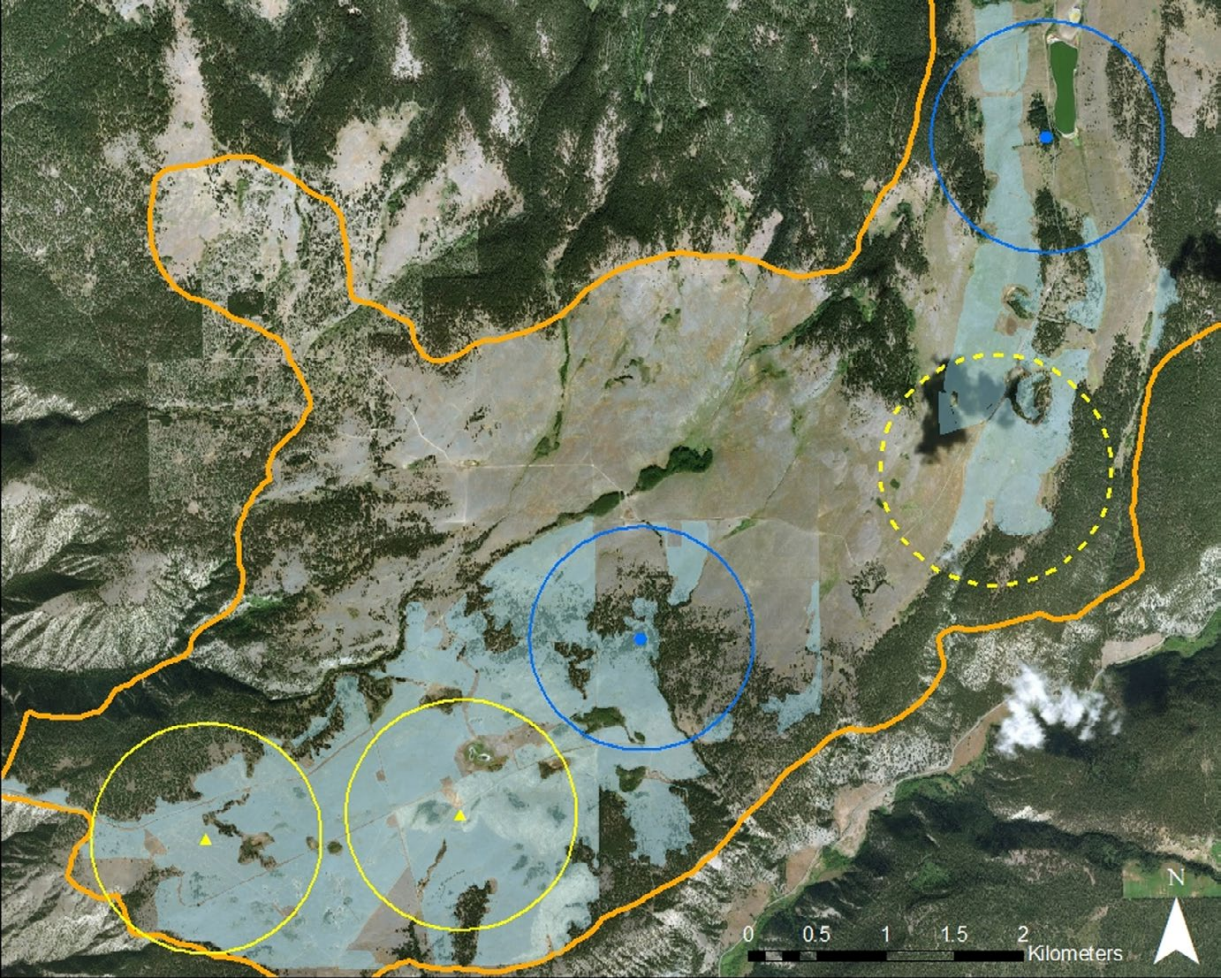
The study area and locations of owl nests on the OK Ranch in 2017. We found two Long‐eared Owl nests (blue circles) and two Short‐eared Owl nests (yellow triangles). The circles encompass 220 ha (radius 840 m), which is approximately half the average distance between neighboring nest locations. The yellow dotted circle represents our best approximation of one nesting territory for Short‐eared Owls, but for which we did not find the actual nest. The goldenrod line outlines the majority of the study area but is truncated in the NE corner. The light blue shading shows areas that had biosolids applied in 2014–2016. Our observations suggested there might have been one additional Short‐eared Owl nest in the open grasslands northwest of the yellow dotted territory, but we did not obtain enough information to localize it. We obtained seen sheet data while driving on main and spur roads as well as some grassy tracks: the main roads are visible as pale lines on the image, but additional smaller routes are not visible at this resolution

The ranch roads were primarily single‐vehicle‐wide dirt or vegetated tracks, with one wider gravel road running through the center of the ranch (visible in Figure 2); travel speeds were 10–30 kph. We standardized our “seen sheet” effort by km driven, but cannot separate effort by treatment because in most locations we were able to see multiple control and biosolids areas at the same time. The ranch has been grazed by cattle for over 100 years and we did not observe any evidence of cows disturbing birds or nests. Terrestrial predators were also present; coyotes (*Canis latrans*) and black bears (*Ursus americanus*) were seen 14 and 28 times, respectively, but other boreal carnivores were observed far less often.

We documented 14 species of raptor, six owl species, and three corvid species (Table S1). These seen sheet data enabled us to locate nests, especially if we saw birds in the same location repeatedly, behaviors such as nest defense, or adult birds carrying prey. We focused on trying to find owl and kestrel nests (Buers et al., 2019). We spent 2–5 hr each in spring and in summer on each of the small open‐forested sites within the study area looking for pellets and Long‐eared Owl nests; sites either had no or very few pellets throughout the entire stand, or we found active nests in trees with many pellets located beneath the nest tree and under nearby roost trees. Three species of diurnal raptor nested on our study area—Northern Harriers, American Kestrels, and Bald Eagles (*Haliaeetus leucocephalus*); we also found nests for Long‐eared and Short‐eared Owls. We analyzed kestrel nest locations, habitat use, and diets previously (Buers et al., 2019), and in this paper, we present key kestrel results for comparison to the other species.

### 2.2 Diets: pellet collection

We dissected regurgitated pellets to determine diets for this suite of species (Marti et al., 2007). Although some authors urge locating nests and then using camera records or direct observation to infer diets, such methods are unable to identify many prey items to species or even genus and records are limited to when birds are using the nests (Bakaloudis et al., 2012; Lewis et al., 2004; Redpath et al., 2001; Robinson et al., 2015; Tornberg & Reif, 2007). In our case, we were interested in identifying which vole and grasshopper species were consumed and if there were changes across time in the prey consumed, including in the postfledging period. Thus, pellets were far more informative than nest observations for our research goals.

At least once a month, we walked ~4 km of fencelines that divided pastures, examining the bases of fenceposts for pellets. Pellets were collected from mid‐April to late August 2017, but wildfires in late July and August restricted field access and extended the time between sampling. We also collected pellets from owl nest sites, paddocks where corvids congregated, and beneath several prominent snags in pastures where we routinely observed perched corvids and kestrels. Pellets were from the current sampling year: in April, we collected only 9 pellets total from our surveys, and all were fresh and intact. In later surveys, the fact we cleared all areas at each survey meant pellets had been deposited after our prior survey.

We preferentially used collection sites to assign pellets to the raptor, owl, or corvid that produced the pellet. Specifically, 100% of Long‐eared Owl pellets were near nests, as were 16% of kestrel pellets, and 6% of Short‐eared Owl pellets; the other Short‐eared Owl pellets were from fencelines near nest sites and were confirmed morphometrically (Holt et al., 1987). Pellets were further identified to species based on similarity to known pellets, including size, shape, texture, and dry mass (Table S2); for example, corvid pellets had a distinct “torpedo” shape with two pointed ends and a flattened bottom. We also compared our material to a reference collection of pellets from captive birds held at the BC Wildlife Park (Kamloops). We could not reliably separate magpie and crow pellets based on size or other attributes, so we grouped them in this study; raven pellets were clearly larger.

### 2.3 Identification of prey within pellets

We identified small mammal remains to species by their dentition and other skull attributes (Heisler et al., 2016; Korpimäki & Norrdahl, 1991; Nagorsen, 2005). If teeth or jaws were not present, we used guard hairs to identify the small mammals to genus if possible (Foresman, 2001). To determine the number of small mammal remains within each pellet, any pellet containing fur and/or up to one full skull of the same species was considered to contain one rodent. If there were multiple jaws or pelvises, we counted that total as representing the number of prey (e.g., two left mandibles indicated two prey). Most voles were identifiable to one of four species: meadow vole (*Microtus pennsylvanicus*), tundra vole (*M. oeconomus*), montane vole (*M. montanus*), and long‐tailed vole (*M. longicaudus*). In contrast to the owls, bones of prey are more typically digested by Northern Harriers (Holt et al., 1987), and therefore many remains in harrier pellets were identified from fur rather than bone.

Birds were identified from beaks, feet, or feathers. We counted birds by the number of beaks in a pellet; if no beaks were present, we assumed feathers indicated one bird was consumed. Grasshoppers were identified from mandibles (Buers et al., 2019); samples of each species of grasshopper present on the ranch were available as a reference collection (Gaudreault et al., 2019). Grasshoppers were counted by the number of mandibles present, with two mandibles for the same species representing one grasshopper eaten; 6 of the 243 pellets contained grasshopper legs but not mandibles, and we were unable to count individuals or resolve species for these pellets. Beetles were also consumed (present in 25 pellets), but we were unable to identify these to a finer taxonomic level, and we did not count individual remains.

Corvids also consumed vegetation. Within the pellets, we were able to identify Douglas fir needles and soapberry seeds. We also found remains of both grass stems and grass seeds, but we did not identify the grass species consumed.

### 2.4 Statistical analysis

For the seen sheet data, we present both “detections,” that is, the number of times we saw one or more birds of a given species, and “counts,” the sum of all birds seen during all detections of a species (e.g., seeing 14 crows, then seven would yield two detections and a count of 21). We used χ^2^ to compare observations on areas with biosolids and areas without biosolids to the random distribution of birds that would be expected given that 24.6% of the ranch had had biosolids applied.

We present three metrics of diets composition. First, we calculated the proportion of pellets containing each prey type (Absolute Frequency of Occurrence: *n* of prey type/*n* pellets). Second, we calculated the proportion of each prey type within the pellets (Relative Frequency of Occurrence; *n* of prey type/*n* total prey consumed). Third, we calculated the biomass of prey killed, using wet biomass estimates of each prey type (Table S3) and the number of each prey recorded. For plants, we counted each pellet with a given plant type as containing one prey item of that type. We did not estimate biomass of plants consumed, because there is no way to determine what had been consumed or digested. Therefore, our estimates of dietary biomass for corvids are based entirely on the animal portion of their diets.

For dietary breadth and dietary overlap, we were hampered by the remains that could not be identified to species (e.g., pellets with nondiagnostic bones or degraded fur). To use standard niche breadth measurements such as Levins' index (Krebs, 1998), we would need to omit these data or assign remains to species even when the remains were not diagnostic. Instead, we present (a) the number of prey species that comprise more than 5% of a species' diet, and (b) the percentage of each species' diet that consisted of small mammals, birds, Orthoptera, Coleoptera, and vegetation.

## 3 RESULTS

**FIGURE 3:**
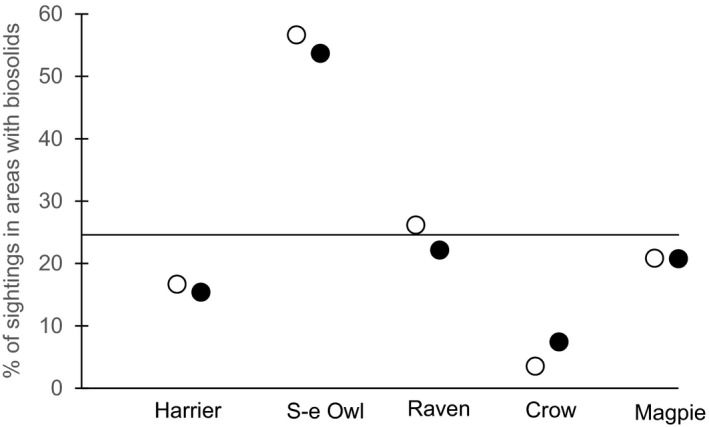
“Seen sheet” observations of Northern Harriers, Short‐eared Owls, and corvids. Values are the % of sightings that occurred on parts of the study area that had had biosolids applications. The horizontal line shows that 24.6% of the study area had biosolids applied prior to the start of our 2017 summer fieldwork; if birds were distributed randomly, this line would form the null expectation for sightings. “S‐e Owl” refers to short‐eared Owls. Long‐eared Owls are not shown because we had one sighting of this nocturnal bird, on an area without biosolids. ● detections of single birds or groups; ○ counts of birds observed

We detected individuals of our focal bird species 342 times, with 755 birds counted, while driving 3,503 km from April to August 2017 (Table 1). Ravens were detected the most often (140 times, 4.0 detections/100 km); we saw Short‐eared Owls the second‐most often, with 95 detections (2.7/100 km). Corvids were typically seen in groups and crows had by far the largest flocks, while raptor sightings often were of single birds. Magpies, ravens, and Northern Harriers did not differentially use or avoid areas with biosolids (Figure 3; all *p* > .63 for χ^2^ tests). Crows avoided areas with biosolids; 7.4% of detections and 3.5% of counts were in these areas (detections χ^2^ = 1.83, *p* = .17; counts χ^2^ = 45.1, *p* < .01). In sharp contrast, Short‐eared Owls had over half of their sightings in areas with biosolids, which is far more than the random expectation (detections χ^2^ = 15.7, *p* < .01; counts χ^2^ = 22.7, *p* < .01). We saw Long‐eared Owls near both nest sites that we located, but we saw only one Long‐eared Owl from a vehicle. We previously reported that we saw American Kestrels 332 times, and they had a strong selection for areas with biosolids, with 59.9% of kestrel observations on treated pastures (Buers et al., 2019).

**TABLE 1:** Observations of six avian species on the OK Ranch in central British Columbia April‐August 2017 in relation to areas where biosolids were applied

	Sites without biosolids			Sites with biosolids			% detections in areas with biosolids	% counts in areas with biosolids
	Detections	Counts	Group size $\bar{x}\;\pm \;$ *SD* (maximum)	Detections	Counts	Group size $\bar{x}\;\pm \;$ *SD* (maximum)		
Crow	25	246	9.8 ± 9.4 (36)	2	9	4.5 ± 4.9 (8)	7.4	3.5
Magpie	42	76	1.8 ± 1.3 (7)	11	20	1.8 ± 1.1 (4)	20.8	20.8
Raven	109	192	1.8 ± 1.6 (13)	31	68	2.2 ± 2.1 (10)	22.1	26.2
Northern Harrier	22	25	1.1 ± 0.4 (2)	4	5	1.3 ± 0.5 (2)	15.4	16.7
Long‐eared Owl^a^	1	1	1 (1)	0	–	–	0	0
Short‐eared Owl^b^	44	49	1.1 ± 0.3 (3)	51	64	1.3 ± 0.5 (3)	53.7	56.6

Note

Because birds were often observed together, we present both detections (all group sizes counted as one detection) and counts (number of birds sighted in total). Birds were not individually identifiable; we are certain we saw many individuals multiple times across the summer. At the beginning of our fieldwork in 2017, biosolids had been spread on 24.6% of the study area.

^a^ We located two nests of Long‐eared Owls (by seeing owls while we were on foot; “seen sheet” data were collected from vehicles). Both were in open forest in areas that had not had biosolids applied.

^b^ We located two nests of Short‐eared Owls and localized the vicinity of one other. Both confirmed nests were in sites where biosolids had been applied.

We located two nests each for Long‐eared owls and Short‐eared Owls. We confirmed successful fledging at one Long‐eared Owl nest, successful hatching at one Short‐eared Owl nest, and failure of one Short‐eared Owl nest; we do not know the fate of the other Long‐eared Owl nest. We also located one nesting area for Short‐eared Owls, but did not locate the actual nest (Figure 2). We used the distance between the adjacent nests (average 1,679 m) to determine ~220 ha as the approximate home range in which pairs would not overlap with the other nesting and territorial owls (this value is similar to previous estimates, Marks et al., 1994). Both Long‐eared Owl nests were within open woodland patches that were surrounded by pastures. Within the 220 ha areas around these nests, 21.0% and 43.3% had been spread with biosolids. For Short‐eared Owls, both confirmed nests were in pastures that had been applied with biosolids. In the 220 ha foraging areas around the two confirmed nests and the nesting area, and biosolids had been spread in 38.2%, 60.3%, and 82.8% (mean 60.4%). Kestrels nested in trees in the woodland patches, with some preference for nearby pastures with biosolids (Buers et al., 2019).

### 3.1 Diets

Short‐eared Owls and Long‐eared Owls ate voles (~92% of prey items and 97% of biomass, from 90 and 46 pellets, respectively, Table 2). For remains we could identify to species, montane voles were the most commonly consumed by both owl species (59.0% for Short‐eared Owls, 100 voles identified; 51.2% for Long‐eared Owls from 41 identified voles). Short‐eared Owls then consumed long‐tailed voles (27.0%) and meadow voles (14.0%), whereas this order was reversed for Long‐eared Owls (meadow voles 26.8%, long‐tailed voles 14.6%), and Long‐eared Owls also ate tundra voles (7.3% of identified vole remains). Mice and grasshoppers were rarely eaten by either species, but Short‐eared Owls also ate 5 individual songbirds.

**TABLE 2:** Diets of Long‐eared Owls and Short‐eared Owls

	Long‐eared Owl^a^					Short‐eared Owl				
	In *n* pellets	*n* prey	AFO	RFO	Biomass (% of diet)	In *n* pellets	*n* prey	AFO	RFO	Biomass (% of diet)
Mammals										
Meadow vole	11	11	23.9	15.5	15.7	14	14	15.6	7.2	7.2
Tundra vole	3	3	6.5	4.2	5.4	0	–	–	–	–
Montane vole	19	21	41.3	29.6	27.1	53	59	58.9	30.4	27.7
Long‐tailed vole	6	6	13.0	8.5	10.7	21	27	23.3	13.9	17.5
*Microtus* spp.	19	24	41.3	33.8	37.9	48	79	53.3	40.7	45.3
Deermouse	1	1	2.2	1.4	0.9	1	1	1.1	0.5	0.3
Jumping mouse	1	1	2.2	1.4	1.0	0	–	–	–	–
Mouse sp.	1	1	2.2	1.4	0.9	0	–	–	–	–
Birds										
Songbird	0	–	–	–	–	4	5	4.4	2.6	1.8
Grasshoppers										
*Camnula pellucida*	0	–	–	–	–	1	7	1.1	3.6	0.06
*Anabrus longipes*	2	2	4.3	2.8	0.31	0	–	–	–	–
Grasshopper spp.	1	1	2.2	1.4	0.05	2	2	2.2	1.0	0.03

Note

For Long‐eared Owls, we had 44 pellets and 71 prey; for Short‐eared Owls, 90 pellets and 194 prey.

Abbreviations: AFO, absolute frequency of occurrence (% of pellets containing each prey type); RFO, relative frequency of occurrence (% of a given prey type out of all prey recorded).

^a^ Four pellets from Long‐eared Owls contained Douglas Fir needles, and one had grass; we suspect these remains were from incidental ingestion while the owl consumed a prey animal, so we have not included them as part of the diet.

Northern Harriers also ate mostly voles; remains were present in 29 of 30 pellets and voles were 38 of 46 prey (Table 3). In 4 cases, we had sufficient bone/dentition remains to identify vole remains to species, and all were montane voles, but the other vole remains consisted mostly of fur and undiagnostic and partially digested bones. Northern Harriers also ate other small mammals (we detected 1 deermouse, *Peromyscus maniculatus*, and 1 chipmunk, *Tamias amoenus*) and songbirds. Kestrels had small mammal remains in 49 of 54 pellets and grasshoppers in 14 of 54 pellets (details in Buers et al., 2019).

**TABLE 3:** Diets of Northern Harriers

	In *n* pellets	*n* prey	AFO	RFO	Biomass (% of diet)
Mammals					
Montane vole	4	4	13.3	8.7	7.7
Deermouse	1	1	3.3	2.2	1.3
Yellow pine chipmunk	1	1	3.3	2.2	3.4
*Microtus* spp.	25	34	83.3	73.9	80.1
Birds					
Vesper Sparrow	5	5	16.7	10.9	7.3
Insects					
*Anabrus longipes*	1	1	3.3	2.2	0.2

Note

We had 30 pellets and identified 46 prey remains.

Abbreviations: AFO, absolute frequency of occurrence (% of pellets containing each prey type); RFO, relative frequency of occurrence (% of a given prey type out of all prey recorded).

The corvids had far more varied diets (Table 4). Ravens consumed voles, grasshoppers, birds, beetles, and vegetation. Grasshoppers dominated the percentage of prey consumed (67.4%), but voles and small mammals dominated the biomass in the diet (62.1%). The clear‐winged grasshopper (*Camnula pellucida*) appeared in only four pellets of 22 sampled, but we detected 88 individual grasshoppers in these four pellets (49.4% of total prey items), resulting in 10.4% of the prey biomass.

**TABLE 4:** Diets of corvids

	Ravens					Crows/Magpies				
	In *n* pellets	*n* prey	AFO	RFO	Biomass^a^ (% of diet)	In *n* pellets	*n* prey	AFO	RFO	Biomass^a^ (% of diet)
Mammals										
Long‐tailed vole	1	1	4.5	0.6	8.5	0	0	–	–	–
*Microtus* spp.	2	2	9.1	1.1	14.4	1	1	1.8	0.3	6.4
Small mammal^b^	6	6	27.3	3.4	39.2	6	6	10.9	1.6	33.3
Birds										
Vesper Sparrow	2	2	9.1	1.1	9.4	0	0	–	–	–
Egg	1	1	4.5	0.6	–	0	0	–	–	–
Insects										
*Anabrus longipes*	3	9	13.6	5.1	6.6	16	49	29.1	13.0	30.5
*Anabrus simplex*	0	0	–	–	–	6	6	10.9	1.6	3.7
*Anabrus* spp.	2	12	9.1	6.7	8.8	0	0	–	–	–
*Camnula pellucida*	4	88	18.2	49.4	10.4	43	214	78.2	56.9	21.5
*Conozoa sulficrons*	2	2	9.1	1.1	0.2	12	15	21.8	4.0	1.2
*Pseudochorthippus curtipennis*	1	2	4.5	1.1	0.1	7	7	12.7	1.9	0.2
*Melanoplus sanguinipes*	0	0	–	–	–	4	9	7.3	2.4	0.5
*Melanoplus bivitattus*	1	1	4.5	0.6	0.2	6	8	10.9	2.1	1.4
*Arphia pseudonietana*	0	0	–	–	–	1	1	1.8	0.3	0.1
*Bruneri brunnea*	0	0	–	–	–	1	1	1.8	0.3	0.1
*Gryllus veletis*	1	5	4.5	2.8	0.5	2	2	3.6	0.5	0.2
Grasshopper spp.	3	6	13.6	3.4	1.3	3	3	5.5	0.8	0.5
Beetle	10	14	45.5	7.9	0.5	11	11	20.0	2.9	0.3
Plants										
*Shepherdia canadensis*	7	7	31.8	3.9	–	31	31	56.4	8.2	–
*Pseudotsuga menziesii*	9	9	40.9	5.1	–	0	–	–	–	–
Grass	11	11	50.0	6.2	–	12	12	21.8	3.2	–

Note

For Common Ravens, we had 22 pellets and 178 prey or plant remains; for Crows/Magpies, 55 pellets and 376 remains.

Abbreviations: AFO, absolute frequency of occurrence (% of pellets containing each prey type); RFO, relative frequency of occurrence (% of a given prey type out of all remains recorded).

^a^ For corvids, we could not estimate the biomass of plants consumed, so the biomasses are based on animal prey.

^b^ These remains were so degraded we could not tell if they derived from vole, mouse, or shrew.

The smaller magpies and crows primarily ate grasshoppers (83.2% of prey, 59.8% of biomass), but also consumed small mammals, beetles, and vegetation. As with ravens, small corvids ate small mammals only rarely (seven prey, 1.9%), but they provided a high percentage of the prey biomass (39.7%). Clear‐winged grasshoppers were by far the most common prey item (56.9%), followed by long‐legged Anabrus (*Anabrus longipes*, 13.0%).

### 3.2 Dietary breadth

The owls and Northern Harriers all focused on voles and had narrow dietary breadths during this breeding season. Both Long‐eared Owls and Short‐eared Owls consumed three species of vole as their main prey. Long‐eared Owls also consumed a few tundra voles (4.2% of prey items, 5.4% of dietary biomass). The corvids consumed many more species than did the owls and harriers, but the number of species that individually were >5% of the diet was small. By this standard, crows and magpies ate three main prey species: clear‐winged grasshoppers, long‐legged Anabrus, and soapberry, whereas for ravens, the dominant prey was clear‐winged grasshoppers, long‐legged Anabrus, and Douglas fir. Additionally, unidentified Coleoptera species and grasses were each more than 5% of the ravens' diet, although these groups likely contained multiple species.

**FIGURE 4:**
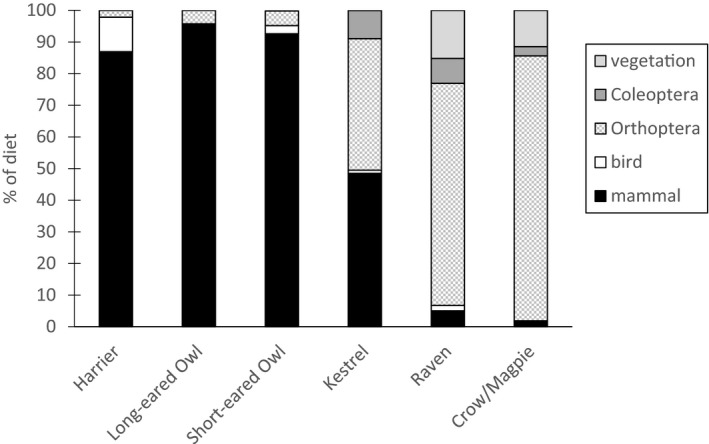
Major prey for seven predatory bird species on a northern grassland in British Columbia. Values are from total number of prey identified for each species (harriers 46, Long‐eared Owls 71, Short‐eared Owls 194, kestrels 101, ravens 178, crows/magpies 376). Mammalian prey consists almost entirely of four species of vole (94%), but also includes four mice, one shrew eaten by a Kestrel, one chipmunk consumed by a Northern Harrier, and 12 prey identifiable only as small mammal. Bird prey included Vesper Sparrows and eggs. Vegetation included grass, Douglas fir, and soapberry. Kestrel data are from Buers et al., (2019)

We also examined dietary niche breadth by considering five groups of prey (Figure 4). Both owl species and Northern Harriers specialized on small mammals (87.0%–95.8%), whereas the corvids had diets dominated by Orthoptera (70.2% for ravens, 83.8% for magpies/crows). Kestrel diets were midway between the corvids and the owls and harriers; 48.5% of their diet was small mammal, and 41.6% was Orthoptera (as analyzed in Buers et al., 2019).

## 4 DISCUSSION

For our first objective, we used seen sheet data to examine whether any of the raptors, owls, or corvids differentially used areas with biosolids. Short‐eared Owls used areas with biosolids more than twice as much as expected by chance; American Kestrels also strongly preferred areas with biosolids (Buers et al., 2019). Both Short‐eared Owls and Long‐eared Owls nested at sites that had high amounts of biosolids‐amended pastures in the surrounding foraging ranges. In contrast, crows strongly avoided areas with biosolids, and magpies, ravens, and Northern Harriers neither selected nor avoided areas with biosolids. These results likely reflect a complex mixture of responses to prey, preference for different amounts of vegetative cover, and interspecific avoidance of other birds. Unfortunately, we do not know if small mammals were more common on areas with biosolids so we cannot fully link these habitat choices to prey; Orthoptera were more abundant on sites with biosolids applications (Gaudreault et al., 2019).

Our second objective, to describe diets of these predatory birds on a northern grassland during a vole peak, showed a clear split in resource use between the two owls and harrier, all of which relied on voles, and the corvids, which primarily consumed Orthoptera and vegetation. Although corvids ate a few voles, plant remains occurred in 74.5% of the crow/magpie pellets and 90.9% of the raven pellets, so we infer that plants were a substantial part of the corvids' biomass intake. Corvids show substantial seasonal variation in their diets and in relation to availability of animal prey (Annala et al., 2012; Temple, 1974), as our results also indicate.

Our third objective was to examine niche breadths and dietary overlaps for these raptors, owls, and corvids. Niche breadths were narrow and nearly identical for both owl species and Northern Harriers, with three vole species comprising almost all of their diets. Corvid diets also showed high overlap with each other, and corvid niche breadths (species that were >5% of the diet) were actually quite small with three prey types for crows and magpies, and five for ravens. Kestrel diets were almost exactly halfway between the owls/harriers and the corvids, as they consisted of about half voles and about half Orthoptera (Buers et al., 2019).

### 4.1 Response of nomadic avian consumers to high prey resources

Our results suggest that the owls cued in on this resource‐rich environment; both species are irruptive nomads that select breeding areas where voles are at high densities (González‐Rojas et al., 2017; Poulin et al., 2001; Selçuk et al., 2017). Short‐eared Owls are strongly nomadic, enabling birds to choose nesting and overwintering sites that have locally abundant prey (Booms et al., 2014; Johnson et al., 2017; Reid et al., 2011). Long‐eared Owls show some nomadism in relation to vole abundance (Korpimäki & Norrdahl, 1991; Tulis et al., 2015), but they are also generalist consumers across their large geographic range (Birrer, 2009; Village, 1981). Similarly, harriers are seminomadic and may seek areas with high vole densities. Thus, the fact that owls chose to breed at the Ranch indicates they detected high vole abundances in spring 2017; we observed no or few owls in later years when small mammals were rare (Meineke, 2020). In this biosolids‐amended grassland landscape during a vole peak, the owls and harriers all specialized on voles. Four species were eaten, but tundra voles were far less commonly consumed than were long‐tailed, montane, and meadow voles. These three species were the main dietary components for both owl species, thus leading to narrow dietary niche breadths and high dietary overlaps during this breeding season. Northern Harriers also relied on voles as prey, but we could identify only a few remains to species due to the degraded bone in these samples.

Despite this dietary convergence, the five Short‐eared owls' and Long‐eared owls' nesting areas were evenly spaced across the ranch, which suggests the nesting pairs were likely hunting in different pastures and avoiding each other's territories. Although this sample size of nesting owls was low, four of the five nests were disproportionately surrounded by biosolids‐amended pastures. This result strongly suggests owls preferred to forage on pastures with biosolids, as does the fact that 51 of 95 (53.6%) detections of Short‐eared Owls were in amended pastures, even though only 24.6% of the area was spread with biosolids. We are less certain about the habitat selection of Northern Harriers, but only 4 of 26 (15.4%) sightings were in amended pastures, thus suggesting that harriers avoided areas with biosolids.

We suspect some of these spatial patterns are due to behavioral responses to each other; Temeles and Wellicome (1992) observed harriers stealing prey from Short‐eared Owls, and both Short‐eared and Long‐eared Owls depredate nestling harriers (Scottish Natural Heritage, 2018). There is also evidence that owls and harriers select specific habitats for hunting. In the eastern United States, Northern Harriers avoided recently mowed areas, but hunted in grasslands, shrublands, and recently burned areas (Massey et al., 2009). In Kentucky, overwintering harriers hunted in denser vegetation than did Short‐eared Owls (Vukovich & Ritchison, 2008). In Illinois, breeding Northern Harriers used taller and denser grasslands than the owls (Herkert et al., 1999). Although we did not specifically tie our observations of owls and harriers to vegetation, our results similarly point to spatial segregation of habitat use despite the high dietary overlap.

### 4.2 Corvid diets and habitat choices

In contrast to the owls and harriers, magpies and ravens did not select or avoid pastures with biosolids, but crows strongly avoided pastures with biosolids. It is not clear to what extent such results reflect selection for given habitats or competitive interactions and behavioral avoidance among the birds (Verbeek & Caffrey, 2002). Corvids could be prey for the larger‐bodied raptors and owls, and Short‐eared Owls defend their breeding areas aggressively (Wiggins et al., 2006).

The corvids had diverse diets despite the abundant voles. Specifically, we identified six Orthopteran species in the diets of ravens (out of 12 identifiable prey types), and 10 Orthoptera out of 15 prey types for the crows and magpies. The primary grasshopper species consumed by corvids, *C. pellucida*, was by far the dominant species we detected during previous grasshopper surveys (Gaudreault et al., 2019). Although the corvids clearly ate more prey species than the owls or harriers, niche breadths were similar in that three to five main prey types provided the majority of the corvids' summer diets.

This reliance on Orthoptera echoes work on American Crows further north in BC, at the Prince George airport; crows there strongly preferred recently mown areas, where grasshoppers were more visible than in longer grass (Kennedy & Otter, 2015). We also observed this selection for habitat over prey abundance, because grasshopper densities were higher on areas with biosolids than areas without (Gaudreault et al., 2019), but crows preferred to forage on pastures without biosolids. This result likely indicates a preference for the sparser grass growth itself.

## 5 CONCLUSIONS

The strong spatial separation of nesting and hunting areas maintained by each nesting pair of birds may be critical for maintaining diverse assemblages of top predators in a prey‐rich ecosystem, even when the predators' diets converge on a few prey types. It would be useful to conduct similar work over more years, as prey numbers fluctuate, to assess how variable the diets of the birds of prey are in this system. Our nesting and seen sheet data suggest extensive spatial and temporal variation in habitat use among species, even as we detected dietary convergence, with owls and harriers eating vole‐dominated diets, corvids eating Orthoptera and vegetation, and kestrels consuming both voles and Orthoptera. It would be valuable to further explore whether these spatiotemporal choices are driven by prey availability, ease of hunting, habitat preference, or interspecific and intraspecific interactions with other avian predators. Further research is also warranted into whether biosolids increase small mammal density in degraded grasslands, thus potentially supporting a diverse community of birds of prey and species of conservation concern.

## CONFLICT OF INTEREST

We have no conflicts to declare.

## AUTHOR CONTRIBUTIONS

**Arianna E. C. Ormrod:** Data curation (supporting); formal analysis (supporting); investigation (lead); writing‐original draft (lead); writing‐review & editing (supporting). **Kirstie J. Lawson:** Data curation (supporting); investigation (supporting); methodology (supporting); supervision (supporting); writing‐review & editing (supporting). **Francis I. Doyle:** Funding acquisition (equal); investigation (equal); methodology (equal); supervision (equal); writing‐review & editing (supporting). **Karen E. Hodges:** Conceptualization (equal); data curation (equal); formal analysis (equal); funding acquisition (equal); project administration (lead); resources (lead); supervision (equal); writing‐review & editing (equal).

## ETHICAL APPROVAL

Our work did not require animal care approval because we did not handle any birds for this work. We had the landowner's permission to work on the property.

## Supporting information

Supplementary Material

## Data Availability

Data are available on cIRcle, a public repository maintained by the University of British Columbia.
