# Corrigendum

**DOI:** 10.1002/ece3.9826

**Published:** 2023-02-21

In the article by Ormrod et al. (2021), the authors hereby correct two data errors and associated calculations and statements. These errors arose as transcription and copy/paste errors during data entry and analysis. One correction concerns misidentification of three vole remains in diets of Long‐eared Owls; the other involves mistakenly omitting a number of detections of Northern Harriers.

**1. Change in diet data for Long‐eared Owls.** The owls did not eat three tundra voles (*Microtus oeconomus*); the study area is not in the geographic range of tundra voles and we confirmed from the original jawbones in the owl pellets that two were *M. montanus* and one was too degraded to confirm to vole species (likely *M. montanus* but counted as unknown vole in our analyses). We also had 46 pellets and 72 prey, not 44 and 71. We have corrected Table 2 and the associated sentences.

**Corrected sentences are as follows:**

***2.3 Identification of Prey within Pellets*** (first paragraph)

“Most voles were identifiable to one of three species: meadow vole (*Microtus pennsylvanicus*), montane vole (*M. montanus*), and long‐tailed vole (*M. longicaudus*).”

***3.1 Diets*** (first paragraph)

“For vole remains we could identify to species, montane voles were the most commonly consumed by both owl species (59.0% for Short‐eared Owls, 100 voles identified; 58.5% for Long‐eared Owls from 41 identified voles). Short‐eared Owls then consumed long‐tailed voles (27.0%) and meadow voles (14.0%), whereas this order was reversed for Long‐eared Owls (meadow voles 26.8%, long‐tailed voles 14.6%).”

***3.2 Dietary breadth*** (first paragraph)

This sentence should be cut because it is wrong: “Long‐eared Owls also consumed a few tundra voles (4.2% of prey items, 5.4% of dietary biomass).”

***4.1 Response of nomadic avian consumers to high prey resources*** (first paragraph)

“Three vole species (long‐tailed, montane, and meadow) were the main prey for both owl species, thus leading to narrow dietary niche breadths and high dietary overlaps during this breeding season.”

***Figure 4 caption***

“Long‐eared owls 72” and “Mammalian prey consists almost entirely of three species of vole (94%)”.

***Table S3***

The line for biomass for tundra voles is accurate but is not needed in this paper; the line should be cut.

**2. Change in seen sheet data for Northern Harriers.** We accidentally omitted a number of sightings during our analysis; we detected these birds 124 times, not 26 times. Two sightings were in March 2017, thus are not included in Table 1 or Figure 3, but these two records are included in the Supplemental Material.

**TABLE 1:** Observations of six avian species on the OK Ranch in Central British Columbia April–August 2017 in relation to areas where biosolids were applied. Because birds were often observed together, we present both detections (all group sizes counted as one detection) and counts (number of birds sighted in total). Birds were not individually identifiable; we are certain we saw many individuals multiple times across the summer. At the beginning of our fieldwork in 2017, biosolids had been spread in 24.6% of the study area.

	Sites without biosolids			Sites with biosolids				
	Detections	Counts	Group size $\bar{x}$ ± SD (maximum)	Detections	Counts	Group size $\bar{x}$ ± SD (maximum)	% detections in areas with biosolids	% counts in areas with biosolids
Crow	25	246	9.8 ± 9.4 (36)	2	9	4.5 ± 4.9 (8)	7.4	3.5
Magpie	42	76	1.8 ± 1.3 (7)	11	20	1.8 ± 1.1 (4)	20.8	20.8
Raven	109	192	1.8 ± 1.6 (13)	31	68	2.2 ± 2.1 (10)	22.1	26.2
Northern Harrier	92	108	1.2 ± 0.4 (3)	30	32	1.1 ± 0.3 (2)	24.6	22.9
Long‐eared Owl^a^	1	1	1 (1)	0	–	–	0	0
Short‐eared Owl^b^	44	49	1.1 ± 0.3 (3)	51	64	1.3 ± 0.5 (3)	53.7	56.6

^a^ We located two nests of Long‐eared Owls (by seeing owls while we were on foot; “seen sheet” data were collected from vehicles). Both were in open forests in areas that had not had biosolids applied.

^b^ We located two nests of Short‐eared Owls and localized the vicinity of one other. Both confirmed nests were in sites where biosolids had been applied.

**FIGURE 3:**
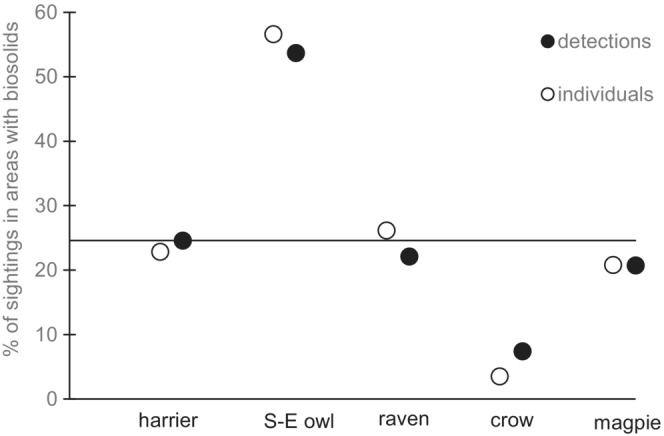


**TABLE 2:** Diets of Long‐eared Owls and Short‐eared Owls. For Long‐eared Owls, we had 46 pellets and 72 prey; for Short‐eared Owls, 90 pellets and 194 prey. AFO: absolute frequency of occurrence (% of pellets containing each prey type); RFO: relative frequency of occurrence (% of a given prey type out of all prey recorded).

		Long‐eared Owl^a^					Short‐eared Owl			
	in *n* pellets	*n* prey	AFO	RFO	Biomass (% of diet)	in *n* pellets	*n* prey	AFO	RFO	Biomass (% of diet)
Mammals										
Meadow vole	11	11	23.9	15.5	16.1	14	14	15.6	7.2	7.2
Montane vole	21	24	45.6	33.8	31.9	53	59	58.9	30.4	27.7
Long‐tailed vole	6	6	13.0	8.5	11.0	21	27	23.3	13.9	17.5
*Microtus* spp.	19	25	41.3	35.2	38.6	48	79	53.3	40.7	45.3
Deer mouse	1	1	2.2	1.4	0.9	1	1	1.1	0.5	0.3
Jumping mouse	1	1	2.2	1.4	1.0	0	–	–	–	–
Mouse sp.	1	1	2.2	1.4	0.9	0	–	–	–	–
Birds										
Songbirds	0	–	–	–	–	4	5	4.4	2.6	1.8
Grasshoppers										
*Camnula pellucida*	0	–	–	–	–	1	7	1.1	3.6	0.06
*Anabrus longipes*	2	2	4.3	2.8	0.31	0	–	–	–	–
Grasshopper spp.	1	1	2.2	1.4	0.05	2	2	2.2	1.0	0.03

^a^ Four pellets from Long‐eared Owls contained Douglas Fir needles, and one had grass; we suspect these remains were from incidental ingestion while the owl consumed a prey animal, so we have not included them as part of the diet.

**Corrected sentences are as follows:**

***4.1 | Response of nomadic avian consumers to high prey resources (second paragraph)***

“In contrast, Northern Harriers neither selected nor avoided pastures with biosolids.”

The authors apologize for the errors.
