# Supplementary material for: Niche partitioning of avian predators in northern grasslands amended by biosolids
Source: Ecol Evol. 2021 May 1;11(11):6248–59. doi: 10.1002/ece3.7461 (PMC8207157; doi:10.1002/ece3.7461)
Supplement: Supplementary file 1 — Supplementary Material [file ECE3-11-6248-s001.docx]

Ormrod et al. Supplemental Online Material

**Table S1.** The corvid, owl, and raptor community observed at the OK Ranch in 2017^a^**.** The data are numbers of detections from seen sheets, i.e. observations we made from vehicles as we drove in the study area. Groups of the same species are counted as one detection. Nests were discovered incidentally or from walking surveys within the small wooded areas, looking for birds, nests and pellets.

| **Common name** | **Latin name** | **Detections** | **Nesting**^b^ | **Dietary analysis** |
| --- | --- | --- | --- | --- |
| American Crow | *Corvus brachyrhynchos* | 27 | inferred | this study |
| Black-billed Magpie | *Pica hudsonia* | 53 | inferred | this study |
| Common Raven | *Corvus corax* | 140 | inferred | this study |
| Short-eared Owl | *Asio flammeus* | 95 | 2 nests, 2 nest areas | this study |
| Long-eared Owl | *Asio otus* | 1 | 2 nests | this study |
| Great Gray Owl | *Strix nebulosa* | 3 | suspected | -- |
| American Kestrel | *Falco sparverius* | 333 | 14 nests | Buers et al. 2019 |
| Northern Harrier | *Circus hudsonius* | 124 | 1 nest nearby | this study |
| Red-tailed Hawk | *Buteo jamaicensis* | 88 | likely nearby | -- |
| Rough-legged Hawk | *Buteo lagopus* | 39 | unlikely | -- |
| Bald Eagle | *Haliaeetus leucocephalus* | 17 | 1 nest | -- |
| Golden Eagle | *Aquila chrysaetos* | 11 | likely nearby | -- |
| Merlin | *Falco columbarius* | 5 | 1 nest nearby | -- |
| Sharp-shinned Hawk | *Accipiter striatus* | 5 | unknown | -- |
| Peregrine Falcon | *Falco peregrinus* | 4 | nearby, on river canyon | -- |
| Turkey Vulture | *Cathartes aura* | 4 | unknown | -- |
| Cooper’s Hawk | *Accipiter cooperii* | 2 | unknown | -- |
| Northern Goshawk | *Accipiter gentilis* | 2 | unknown | -- |
| Prairie Falcon | *Falco mexicanus* | 2 | unknown | -- |

^a^In 2016, we had one sighting each of Great Horned Owl, Screech Owl (roadkill), Barred Owl, Flammulated Owl, and Osprey (*Pandion haliaetus*). In 2017, we heard but did not see Great Horned Owls, Barred Owls, and Flammulated Owls. We suspect these owls nested in denser forests adjacent to the focal grassland – open woodland study area.

^b^ We “inferred” reproduction for species with multiple sightings throughout the summer or when we saw juveniles in late summer. We classified reproduction as “unlikely” for migrants, and “unknown” for birds that should have been readily detectable (diurnal raptors) but that we saw <10 times. For Great Gray Owls and Great Horned Owls, we “suspected” there was reproduction based on hearing them and seeing some Great Gray Owls, but nests may have been in forests located outside our study area. Finally, we strongly suspect Golden Eagles and Red-tailed Hawks bred near our study area, but not on it; we think we would have detected nest defense had nests been on our study area.

**Table S2.** Attributes of the pellets by bird species. Mass, length, and width values are means ± 1 SD (range) of complete pellets; for shape, we provide the percentage of pellets of each kind derived from both full and partial pellets. Partial pellets could be identified to species based on where the pellet was found (e.g. nest site) or when the measurements (e.g. maximum width) and shape combined to be diagnostic.

|  | measurements (complete pellets only) | | | shape^a^ (% of all pellets) | | |
| --- | --- | --- | --- | --- | --- | --- |
| species (n complete pellets, n pellets total) | dry mass (g) | length (mm) | width (mm) | torpedo | oval | irregular |
| Long-eared Owl^b^ (36, 46) | 2.12±0.91 | 39.4±7.4 | 20.0±2.5 | 47.8 | 41.3 | 6.5 |
| Short-eared Owl (81, 90) | 3.37±1.38 | 47.0±8.7 | 20.8±2.7 | 68.9 | 24.4 | 6.7 |
| Northern Harrier (21, 30) | 2.98±1.00 | 40.6±8.4 | 16.8±6.4 | 56.7 | 40.0 | 3.3 |
| Common Raven (18, 22) | 1.47±0.59 | 33.1±4.9 | 18.0±2.4 | 85.7 | 9.5 | 4.8 |
| American Crow/ Black-billed Magpie (44, 55) | 0.36±0.16 | 20.8±3.7 | 11.4±1.7 | 74.5 | 21.8 | 3.6 |
| American Kestrel^c^ (89, 118) | 0.44±0.18 | 21.2±5.1 | 11.5±2.6 | 0.9 | 73.7 | 14.4 |

^a^**Torpedo:** Pellet was cylindrical, with one or both ends pointed. For owls, one end was often rounded and the pointed part of the pellet was generally a hair tuft indicating direction of regurgitation, i.e. the last part of the pellet to be egested. Corvid pellets with a torpedo shape often had both ends pointed and a flattened side.

**Oval:** Pellet was spherical to cylindrical with rounded ends.

**Irregular:** Pellet that was older or had been damaged. These pellets were partially torn open, disintegrating, or flattened.

^b^For Long-eared Owls, we also had 2 prey remains that were skulls with tissue of small mammals for 4.3% of the total.

^c^ For Kestrels, 13 remains were skulls with tissue, for 11.0% of the total. Dietary results for Kestrels are in Buers et al. 2019.

**Table S3.**  Average mass of prey used to calculate biomass in owl and corvid diets. We used BC sources for mammals, but for insects we could not find local estimates. We developed a version of this table for species from the same study area that were eaten by kestrels (Buers et al. 2019). Here, we present species eaten by the owls and corvids. Values are wet masses.

| **Species** | **Common Name** | **Body mass (g)** | **Source** |
| --- | --- | --- | --- |
| **Mammals** |  |  |  |
| *Microtus pennsylvanicus* | Meadow vole | 34.9 | Nagorsen 2005 |
| *Microtus longicaudus* | Long-Tailed vole | 43.7 | Nagorsen 2005 |
| *Microtus montanus* | Montane vole | 31.6 | Nagorsen 2005 |
| *Microtus oeconomus* | Tundra vole | 44.2 | Nagorsen 2005 |
| *Peromyscus maniculatus* | Deer mouse | 21.7 | Nagorsen 2005 |
| *Zapus hudsonicus* | Western jumping mouse | 24.6 | Nagorsen 2005 |
| *Tamias amoenus* | Yellow pine Chipmunk | 55.0 | Nagorsen 2005 |
| *Microtus* spp. | mean of 4 *Microtus* | 36.9 | -- |
| Small mammal | mean of *Microtus* and 2 mice | 33.45 | -- |
|  |  |  |  |
| **Birds** |  |  |  |
| *Pooecetes gramineus* | Vesper Sparrow | 24.0 | Jones and Cornely 2002 |
|  |  |  |  |
| **Insects** |  |  |  |
| *Anabrus longipes* | Long-legged Anabrus | 3.75 | DeFoliart et al. 1982 |
| *Anabrus simplex* | Mormon cricket | 3.75 | DeFoliart et al. 1982 |
| *Arphia pseudonietana* | Red-winged grasshopper | 0.328 | Capinera and Thompson 1987 |
| *Bruneri brunnea* | Bruner slant-faced grasshopper | 0.376 | Larsen et al. 1988-94 |
| *Camnula pellucida* | Clear-winged grasshopper | 0.605 | Pfadt 1994 |
| *Conozoa sulcifrons*^a^ | Groove-headed grasshopper | 0.483* | -- |
| *Gryllus veletis* | Spring field cricket | 0.488 | Cade and Wyatt 1984 |
| *Melanoplus bivittatus* | Two-striped grasshopper | 1.086 | Bird 1966 |
| *Melanoplus sanguinipes* | Migratory grasshopper | 0.313 | Fielding 2004 |
| *Pseudochorthippus curtipennis* | Meadow grasshopper | 0.19 | Rode 2017 |
| *Meloe spp.* | Oil beetle | 0.180 | Saul-Gershenz 2017 |

^a^ We could not find mass data for this species. We used the mean from the other grass-eating grasshoppers.

***Literature cited***

Bird, R.D., Allen, W. and Smith, D.S. 1966. The responses of grasshoppers to ecological changes produced by agricultural development in southwestern Manitoba. Can. Entomol. – 98: 1191-1205.

Cade, W.H. and Wyatt, D.R. 1984. Factors affecting calling behaviour in field crickets, Teleogryllus and Gryllus (age, weight, density, and parasites). Behaviour – 88: 61-75.

Capinera, J. L. and Thompson, D.C. 1987. Dynamics and structure of grasshopper assemblages in shortgrass prairie. Can. Entomol. – 119: 567-575.

DeFoliart, G. R., M. D. Finke, and M. L. Sunde. 1982. Potential value of the Mormon Cricket
 (Orthoptera: Tettigoniidae) harvested as a high protein feed for poultry. J. Econ. Entomol. – 75: 848-852.

Fielding, D. J. 2004. Developmental time of *Melanoplus sanguinipes* (Orthoptera: Acrididae) at high latitudes. J. Physiol. Ecol. – 33: 1513-1522.

Jones, S. L. and J. E. Cornely. 2002. Vesper Sparrow (*Pooecetes gramineus*), version 2.0. In: Poole, A. F. and Gill, F. B. (eds.), The Birds of North America. Cornell Lab of Ornithology. <https://doi.org/10.2173/bna.624>

Larsen, J. C., Hutchason, J.A., McNary, T., and Zimmerman, K. 1988-94. The Wyoming grasshopper information system. Cooperative Agricultural Pest Survey. University of Wyoming, Laramie.

Mole, S. and Zera, A.J. 1993. Differential allocation of resources underlies the dispersal-reproduction trade-off in the wing-dimorphic cricket, *Gryllus rubens*. Oecologia – 93: 121-127.

Nagorsen, D. W. 2005. Rodents and Lagomorphs of British Columbia. Royal British Columbia Museum, Victoria.

Pfadt, R.E. 1994. Field Guide to Common Western Grasshoppers. Wyoming Agricultural
Experiment Station. Riverdale, MD.

Rode, M., Lemoine, N.P., and Smith, M.D. 2017. Prospective evidence for independent nitrogen and phosphorus limitation of grasshopper (*Chorthippus curtipennis*) growth in a tallgrass prairie. PLoS One 12: 1-12.

Saul-Gershenz, L. 2017. Host range evolution of cleptoparasite *Meloe franciscanus* (Coleoptera: Meloidae). PhD Dissertation, University of California Davis.
